# Incongruent Treatment Recommendations Between Left and Right Hip Bone Mineral Densities

**DOI:** 10.7759/cureus.69923

**Published:** 2024-09-22

**Authors:** Abhilasha Singh, Anu Sharma

**Affiliations:** 1 Endocrinology, Diabetes, and Metabolism, East Valley Diabetes and Endocrinology, San Tan Valley, USA; 2 Endocrinology, Diabetes, and Metabolism, University of Utah Health, Salt Lake City, USA; 3 Endocrinology, Diabetes, and Metabolism, University of Florida, Gainesville, USA

**Keywords:** bone mineral density, difference in bone density at hip, dxa, frax, osteoporosis

## Abstract

Objective

The official position of the International Society for Clinical Densitometry (ISCD) is that either hip site can be scanned with dual-energy X-ray absorptiometry (DXA) imaging. Whether there is a difference in guideline-based treatment recommendations between hip sites has not previously been assessed.

Methods

We conducted a retrospective analysis of all individuals who underwent DXA imaging at a single center from September 1, 2018, to October 31, 2019. Women aged ≥ 40 years old and men aged ≥ 50 years old were included. Individuals without bilateral hip measurements or who were on osteoporosis treatment were excluded. The 10-year probability of fracture using the fracture risk assessment tool (FRAX) was calculated for each hip when the worst T-score was in the osteopenia range.

Results

Of the included participants, 10% (n = 151/1505) had incongruent treatment recommendations (ITR) between the left and right hips. In the ITR group, 70% (n = 106) had osteopenia and 30% (n = 45) had osteoporosis. Age was the only significant risk factor for ITR overall (OR: 1.06, 95% CI: 1.04-1.09). In the osteopenia group, however, age (OR: 1.17, 95% CI: 1.13-1.21), history of parental hip fracture (OR: 3.16, 95% CI: 1.65-6.05), and glucocorticoid use (OR: 4.18, 95% CI: 1.6-11.0) were associated with ITR. In the osteoporosis group, the right hip (femoral neck and total) T-scores were significantly lower compared to the left.

Conclusion

Measuring bone mineral density on both hips changes treatment recommendations in 10% of people. This was more likely in those with osteopenia who were older, had a parental history of hip fracture, or were on glucocorticoids. Measuring both hips did not add time or cost to patient care. Given the minimal disadvantages to measuring both hips, there should be a strong consideration to change the current guidance of only measuring one hip.

## Introduction

Dual-energy X-ray absorptiometry (DXA) remains the modality of choice for the diagnosis of osteoporosis. The current International Society for Clinical Densitometry (ISCD) guideline recommends measuring bone mineral density (BMD) at the spine, and a single hip site with measurement of the forearm under certain clinical circumstances, e.g., history or hyperparathyroidism, or if hip or spine cannot be interpreted [1,2]. As the recommendation does not include bilateral hip BMD measurement routinely [3], individuals undergoing unilateral hip imaging may be at risk for underdiagnosis if a possible difference in BMD/T-score exists between both hips. Indeed, prior studies have noted a discordance in BMD at hip sites. Despite this, there has been a consistent recommendation that either hip can be imaged due to the high correlation between right and left hip BMD. The studies supporting unilateral hip BMD measurement focused on the actual BMD values [4-7] and did not truly reflect the clinician’s incorporation of risk factors that may change treatment recommendations when T-scores are in the osteopenic range.

We hypothesized that there is an incongruence in clinical treatment recommendations between the left and right hip BMD and patients undergoing unilateral hip imaging may be at risk for underdiagnosis. We aimed to compare T-scores and calculated 10-year probabilities of fracture using the fracture risk assessment tool (FRAX) [8] at both hips to evaluate if there would be differing treatment recommendations between hips based on the current guidelines for osteoporosis [9-12].

## Materials and methods

After the institutional review board's approval (IRB#00127038) and in accordance with the Helsinki Declaration on the ethical standards for research conduct, individuals undergoing DXA at a single center from September 1, 2018, to October 31, 2019, were identified from the clinical enterprise data warehouse (EDW) [13]. Pre-specified variables for inclusion included post-menopausal women of age ≥ 40 years old and men aged ≥ 50 years old at the time of bone density measurement. Post-menopausal status was verified by the patient report. Exclusion criteria were age < 40 years in women and age < 50 years in men, inability to measure bone density at both hips, and current or prior osteoporosis treatment (including oral or intravenous bisphosphonates, denosumab, teriparatide, abaloparatide, or romosozumab). Demographic data and clinical variables were extracted from the EDW and subsequently confirmed by chart review. Each patient completed a questionnaire before DXA scanning, which is part of the routine clinical care at this clinic site. Clinical data extracted from this questionnaire were age at the time of measuring BMD, sex, body mass index (BMI) at the time of measuring BMD, history of rheumatoid arthritis, prior or current glucocorticoid use (defined as exposure to prednisone 5 mg daily or equivalent for three months or more), menopausal age in women, personal history of fracture, parental history of hip fracture, presence of risk factors for secondary osteoporosis (type 1 diabetes mellitus, osteogenesis imperfecta, untreated hyperthyroidism, hypogonadism, primary ovarian insufficiency (age < 45 years), chronic malnutrition, malabsorption, or chronic liver disease), current smoking status, and alcohol use of three or more units per day. All individuals underwent BMD measurement with the same machine (Horizon W S/N 301623 M (Hologic, Inc., Marlborough, MA) utilizing fast array mode). BMD and T-scores at the lumbar spine (L1-L4), right total hip, left total hip, right femoral neck, left femoral neck, and distal radius (if performed) were recorded. T-scores were obtained from the manufacturer’s database for the lumbar spine and distal radius. T-scores were obtained from the National Health and Nutrition Examination Survey (NHANES) III data for the femoral neck and total hip. Definitions of normal BMD, osteopenia, and osteoporosis were defined as T-score > -1 as normal, between -1 and -2.5 as osteopenia, and < -2.5 as osteoporosis, consistent with the World Health Organization (WHO) criteria [5].

**Figure 1:**
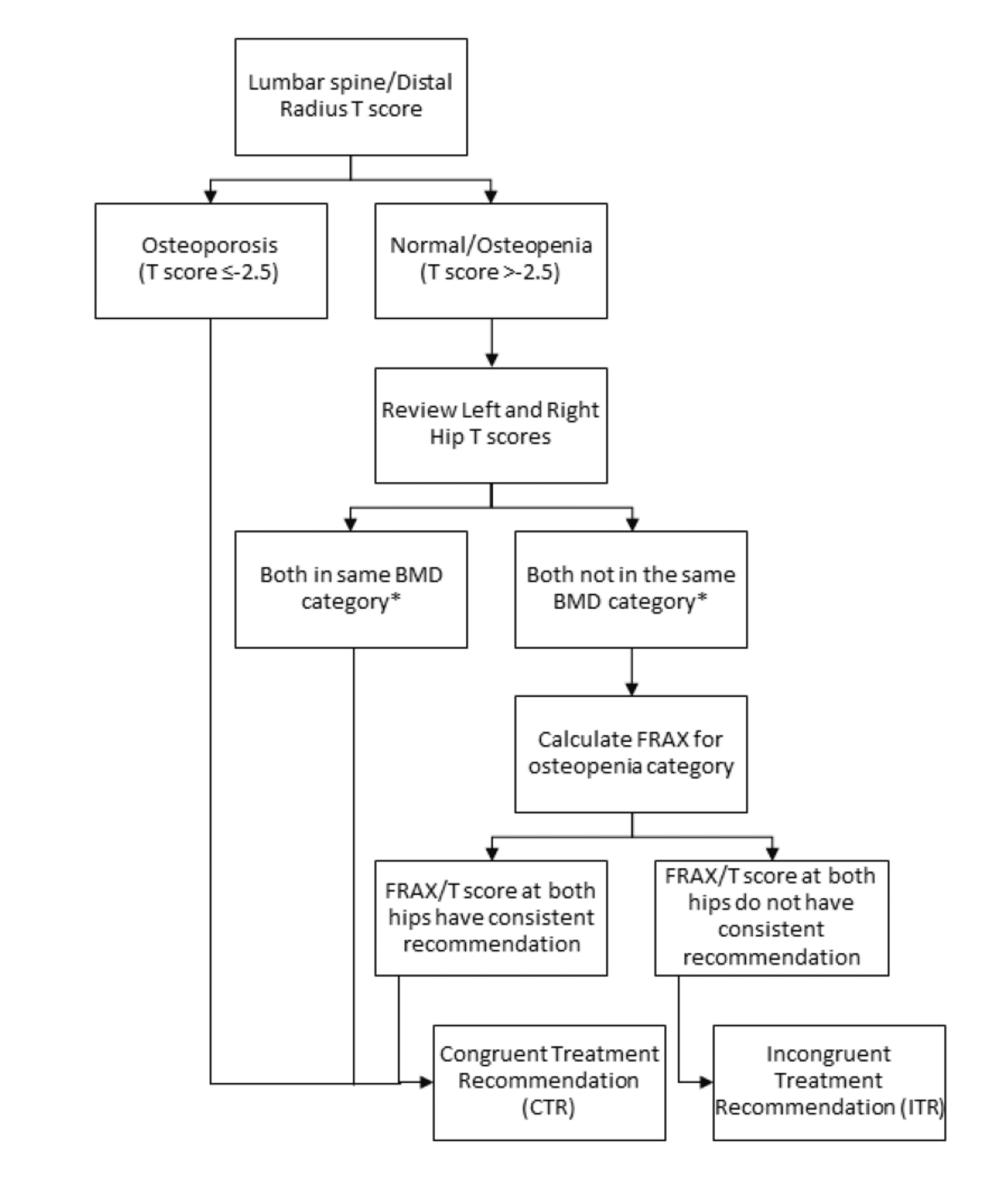
Flow diagram depicting criteria for group assignment. * Bone mineral density (BMD) categories were defined as T-score > -1 as normal, between -1 and -2.5 as osteopenia, and < -2.5 as osteoporosis. FRAX: fracture risk assessment tool.

A congruent treatment recommendation (CTR) was defined as the lumbar spine or distal radius T-score in the osteoporosis range regardless of hip scores, or both hip sites achieving the same treatment recommendation either by the T-score or FRAX calculation as indicated in the current guidelines (Figure 1) [9-11]. An incongruent treatment recommendation (ITR) was defined as a T-score > -2.5 in the lumbar spine and distal radius plus either (i) osteoporosis T- score in one hip and osteopenia/normal T-score in the contralateral hip not meeting treatment criteria per FRAX or (ii) osteopenia T-score meeting treatment criteria per FRAX in one hip but osteopenia/normal T-score in the contralateral hip not meeting treatment criteria per FRAX (Figure 1). Treatment criteria per FRAX were defined as ≥20% risk for a major osteoporotic fracture and/or ≥3% risk for a hip fracture [9].

Statistical analysis

Statistical analysis was performed using SPSS version 27 software (IBM Corp., Armonk, NY). GraphPad version 7 (GraphPad Software, San Diego, CA) was used for the graphical representation of data. Categorical variables were reported as percentages and analyzed with Pearson's chi-squared or Fisher’s exact test when applicable. Continuous variables were expressed as mean with standard deviation (SD) when normally distributed and subjected to the two-sample t-test. Continuous variables with a non-Gaussian distribution were reported as median with interquartile range (25th-75th IQR) and analyzed with non-parametric testing (Wilcoxon rank sum test). Multivariable analysis and calculation of odds ratios were conducted to determine the effect of clinical variables on finding differing diagnoses at each hip site. Sensitivities and specificities of both the right and left were also determined when applicable. Differences with a significance level of p < 0.05 were considered to be statistically significant.

## Results

Clinical characteristics

**Figure 2:**
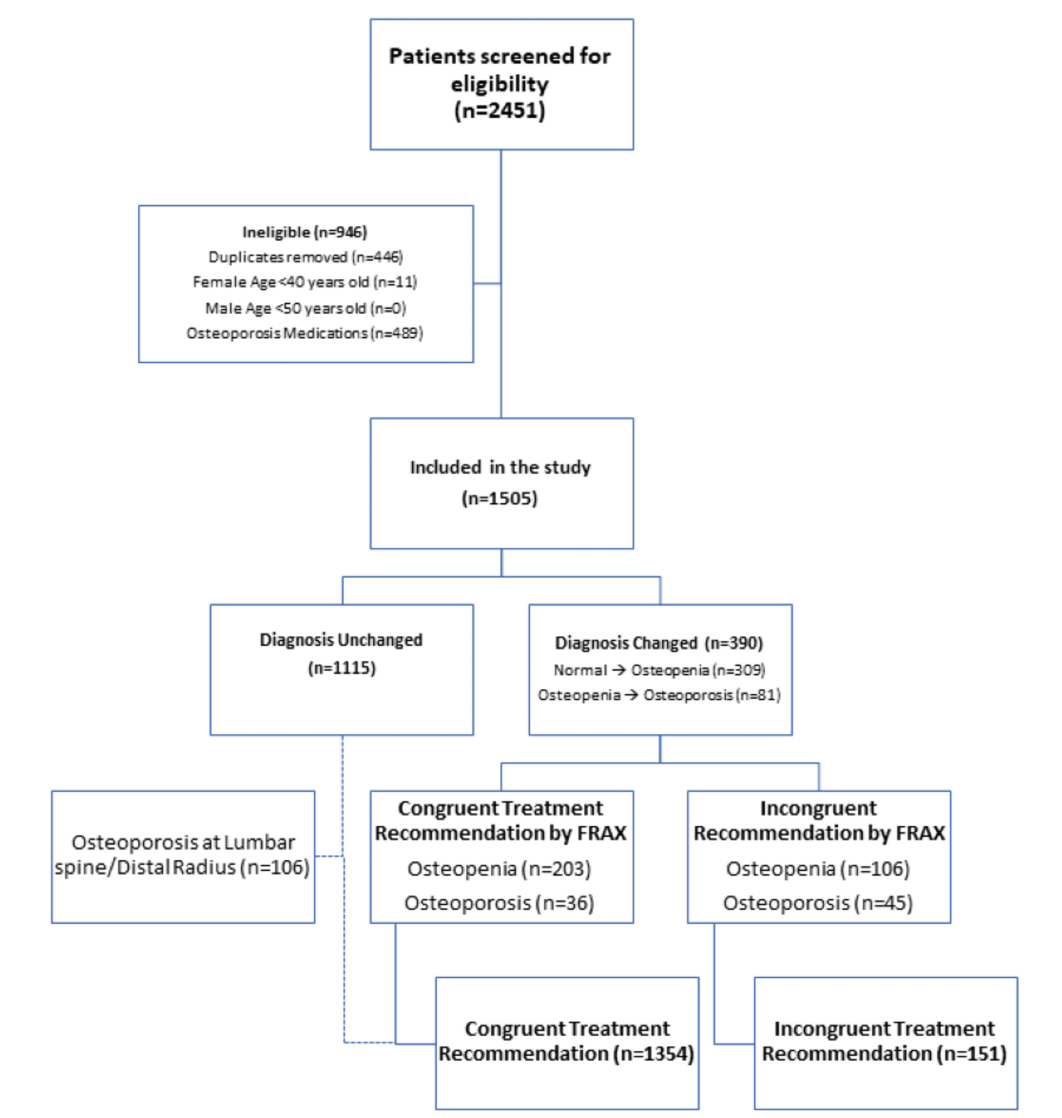
Flow diagram of cohort composition. FRAX: fracture risk assessment tool.

After reviewing 2451 individual patient charts, 946 were deemed ineligible and 1505 were included in the final analysis (Figure 2). Of the participants, 10% (n = 151) had ITR, with most receiving a final diagnosis of osteopenia by T-score (70%, n = 106). Individuals with ITR were older (ITR age = 72 ± 8 years vs. CTR age = 67 ± 9 years, p < 0.01) and were more likely to report glucocorticoid use (ITR 13% vs. CTR 8.4%, p = 0.04) compared to the CTR group (Table 1).

**Table 1:** Characteristics of individuals undergoing bone density measurement with congruent and incongruent treatment recommendations at hip sites. Alcohol use was defined as ingesting three or more alcohol units per day. Smoking was defined as currently smoking at the time of the questionnaire. Categorical variables were analyzed with Pearson's chi-squared or Fisher’s exact test when applicable. Continuous variables were analyzed with two-sample t-test. BMI: body mass index; Hx: history; RA: rheumatoid arthritis.

Patient characteristics	Congruent treatment recommendation (n = 1354)	Incongruent treatment recommendation (n = 151)	p-value
Age (years)	67 ± 9	72 ± 8	<0.01
Female sex, n (%)	1137 (84)	130 (86)	0.5
Caucasian, n (%)	1226 (90.6)	138 (91.3)	0.3
Black, n (%)	11 (0.8)	1 (0.7)	0.3
Hispanic, n (%)	28 (2.0)	1 (0.7)	0.3
Asian, n (%)	31 (2.3)	3 (2.0)	0.3
Native American/Pacific Islander, n (%)	7 (0.5)	3 (2.0)	0.3
Others, n (%)	51 (3.8)	5 (3.3)	0.3
Age at menopause (years)	48 ± 6	48 ± 7	0.8
BMI (kg/m2)	27 ± 6	26 ± 6	0.7
Personal Hx of fracture, n (%)	166 (12)	23 (15)	0.3
Parental Hx of hip fracture, n (%)	157 (12)	22 (15)	0.3
Glucocorticoid use, n (%)	113 (8.4)	20 (13)	0.04
RA, n (%)	40 (3.0)	7 (4.6)	0.3
Alcohol use, n (%)	1 (0.1)	0 (0.0)	0.3
Smoking, n (%)	18 (1.3)	2 (1.3)	1.0

Bone densitometry results

**Figure 3:**
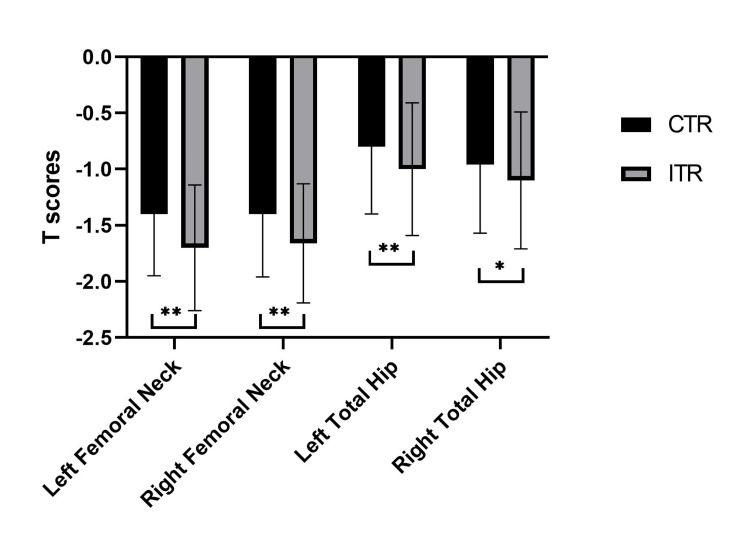
Femoral neck and total hip T-scores in individuals with osteopenia. * p < 0.05; ** p < 0.01. CTR: congruent treatment recommendation; ITR: incongruent treatment recommendation.

While those achieving an osteoporosis diagnosis by T-score were similar between groups (ITR 30% vs. CTR 25%), osteopenia by T-score was more frequent in the ITR group (ITR 70% vs. CTR 55%, p < 0.01). BMD and T-scores were significantly lower in the ITR group at all hip sites compared to the CTR group, likely due to only abnormal T-scores in the ITR group (Table 2). However, these differences persisted in the osteopenia T-score subgroup at all hip sites (Figure 3) but only at the total left hip in the osteoporosis T-score subgroup (Tables 3, 4). The calculated FRAX scores at hip sites when T-scores were in the osteopenia range were higher in the ITR group compared to the CTR group (Table 5). Of the participants, 12% (n = 106) in the osteopenia T-score group had incongruent FRAX treatment recommendations, and 12% (n = 45) in the osteoporosis T-score group had FRAX scores recommending no treatment when the contralateral hip had a T-score of ≤ -2.5.

**Table 2:** Bone densitometry results of individuals undergoing bone density measurement with congruent and incongruent treatment recommendations at hip sites. Treatment was recommended by FRAX if 10-year probability of MOF was ≥ 20% and or hip fracture ≥3%. Continuous variables were expressed as mean with standard deviation (SD) when normally distributed and subjected to the two-sample t-test. Differences with a significance level of p < 0.05 were considered to be statistically significant. DXA: dual-energy X-ray absorptiometry; BMD: bone mineral density; FRAX: fracture risk assessment tool; MOF: major osteoporotic fracture.

DXA characteristics	Congruent treatment recommendation (n = 1354)	Incongruent treatment recommendation (n = 151)	p-value
Normal, n (%)	270 (20)	0 (0)	<0.01
Osteopenia, n (%)	752 (55)	106 (70)	<0.01
Osteoporosis, n (%)	332 (25)	45 (30)	<0.01
Lumbar spine BMD	0.93 ± 0.15	0.93 ± 0.12	0.8
Lumbar spine T score	-1.1 ± 1.3	-1.1 ± 1.1	0.6
Right total hip BMD	0.84 ± 0.13	0.79 ± 0.09	<0.01
Right total hip T score	-0.9 ± 1.0	-1.3 ± 0.7	<0.01
Left total hip BMD	0.86 ± 0.1	0.81 ± 0.08	<0.01
Left total hip T score	-0.8 ± 1.0	-1.1 ± 0.6	<0.01
Right femoral neck BMD	0.70 ± 0.12	0.65 ± 0.07	<0.01
Right femoral neck T score	-0.1.4 ± 1.0	-1.9 ± 0.6	<0.01
Left femoral neck BMD	0.70 ± 0.11	0.65 ± 0.07	<0.01
Left femoral neck T score	-1.4 ± 0.9	-1.8 ± 0.6	<0.01
Distal radius BMD	0.64 ± 0.11	0.64 ± 0.10	0.9
Distal radius T score	-1.2 ± 1.5	-1.1 ± 1.0	0.8
10-year probability of fracture by FRAX - right MOF (%)	10 ± 6	14 ± 5	<0.01
10-year probability of fracture by FRAX - right hip fracture risk (%)	1.7 ± 1.9	3.5 ± 2.4	<0.01
10-year probability of fracture by FRAX - left MOF (%)	10 ± 5	14 ± 5	<0.01
10-year probability of fracture by FRAX - left hip fracture risk (%)	1.8 ± 2.4	3.4 ± 2.4	<0.01
Treatment recommended by FRAX (n, %)	103 (7.6)	106 (70)	<0.01

**Table 3:** Bone densitometry results of individuals undergoing bone density measurement with congruent and incongruent treatment recommendations at hip sites in the osteopenia group. Continuous variables were expressed as mean with standard deviation (SD) when normally distributed and subjected to the two-sample t-test. Differences with a significance level of p < 0.05 were considered to be statistically significant. BMD: bone mineral density.

Anatomical site	Congruent treatment recommendation (n = 752)	Incongruent treatment recommendation (n = 106)	p-value
Lumbar spine BMD	0.93 ± 0.10	0.95 ± 0.13	0.2
Lumbar spine T score	-1.0 ± 0.9	-0.9 ± 1.1	0.1
Right total hip BMD	0.84 ± 0.08	0.82 ± 0.08	0.02
Right total hip T score	-0.96 ± 0.61	-1.1 ± 0.6	0.01
Left total hip BMD	0.86 ± 0.08	0.83 ± 0.08	<0.01
Left total hip T score	-0.8 ± 0.6	-1.0 ± 0.59	<0.01
Right femoral neck BMD	0.70 ± 0.07	0.67 ± 0.06	<0.01
Right femoral neck T score	-1.4 ± 0.56	-1.7 ± 0.53	<0.01
Left femoral neck BMD	0.70 ± 0.07	0.67 ± 0.07	<0.01
Left femoral neck T score	-1.4 ± 0.55	-1.7 ± 0.56	<0.01

**Table 4:** Bone densitometry results of individuals undergoing bone density measurement with congruent and incongruent treatment recommendations at hip sites in the osteoporosis group. Continuous variables (BMD and T-scores) were expressed as mean with standard deviation (SD) when normally distributed and subjected to the two-sample t-test. BMD: bone mineral density.

Anatomical site	Congruent treatment recommendation (n = 332)	Incongruent treatment recommendation (n = 45)	p-value
Lumbar spine BMD	0.78 ± 0.11	0.89 ± 0.10	<0.01
Lumbar spine T score	-2.5 ± 0.96	-1.4 ± 0.8	<0.01
Right total hip BMD	0.71 ± 0.09	0.73 ± 0.07	0.21
Right total hip T score	-1.95 ± 0.74	-1.8 ± 0.52	0.14
Left total hip BMD	0.73 ± 0.10	0.76 ± 0.07	0.03
Left total hip T score	-1.8 ± 0.8	-1.5 ± 0.51	0.03
Right femoral neck BMD	0.59 ± 0.07	0.59 ± 0.05	0.7
Right femoral neck T score	-2.4 ± 0.5	-2.4 ± 0.5	0.8
Left femoral neck BMD	0.6 ± 0.08	0.61 ± 0.05	0.23
Left femoral neck T score	-2.3 ± 0.66	-2.2 ± 0.4	0.18

**Table 5:** Diagnoses were made by T scores at the lumbar spine, right hip, left hip, and distal radius in individuals undergoing bone density measurement with congruent and incongruent treatment recommendations at hip sites. Treatment was recommended by FRAX if the 10-year probability of MOF was ≥ 20% and or hip fracture ≥ 3%. Continuous variables are expressed as mean with standard deviation (SD) when normally distributed and subjected to the two-sample t-test. DXA: dual-energy X-ray absorptiometry; FRAX: fracture risk assessment tool; L-spine: lumbar spine; RTH: right total hip; LTH: left total hip; RFN: right femoral neck; LFN: left femoral neck; MOF: major osteoporotic fracture.

DXA characteristic	Congruent treatment recommendation (n = 1354)	Incongruent treatment recommendation (n = 151)	P-value
L-spine normal, n (%)	532 (45)	50 (39)	<0.01
L-spine osteopenia, n (%)	461 (39)	78 (61)	<0.01
L-spine osteoporosis, n (%)	192 (16)	0 (0)	<0.01
RTH normal, n (%)	701 (52)	44 (29)	<0.01
RTH osteopenia, n (%)	581 (43)	103 (68)	<0.01
RTH osteoporosis, n (%)	72 (5)	4 (3)	<0.01
LTH normal, n (%)	804 (59)	62 (41)	<0.01
LTH osteopenia, n (%)	496 (37)	89 (59)	<0.01
LTH osteoporosis, n (%)	54 (4)	0 (0)	<0.01
RFN normal, n (%)	428 (32)	19 (13)	<0.01
RFN osteopenia, n (%)	757 (56)	101 (67)	<0.01
RFN osteoporosis, n (%)	169 (12)	31 (20)	<0.01
LFN normal, n (%)	441 (32)	15 (10)	<0.01
LFN osteopenia, n (%)	755 (56)	124 (82)	<0.01
LFN osteoporosis, n (%)	158 (12)	12 (8)	<0.01
Treatment recommended by FRAX, right, n (%)	120 (16)	42 (36)	<0.01
Treatment recommended by FRAX, left, n (%)	133 (17)	64 (46)	<0.01

Risk factors for ITR

**Figure 4:**
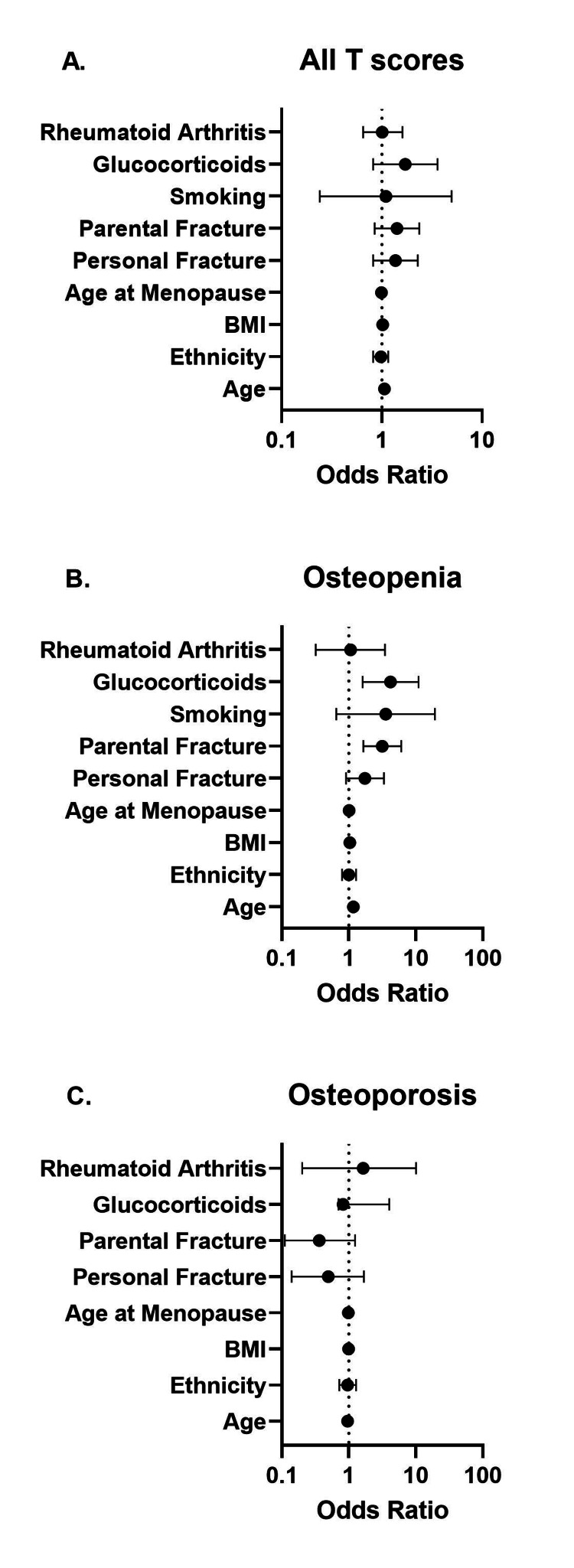
Forrest plot of odds ratios (95% CI) for clinical factors associated with incongruent treatment recommendations in (A) all individuals, (B) individuals with osteopenia, and (C) individuals with osteoporosis. Alcohol was suppressed in all due to the small sample size (n = 1). Smoking was suppressed in osteoporosis due to collinearity. BMI: body mass index.

On multivariable analysis, age was the only significant risk factor for ITR (OR: 1.06, 95% CI: 1.04-1.09). Utilizing the receiver operating characteristic curve’s optimal threshold, age ≥ 68 years was 69% sensitive in determining if ITR would be present (area under the curve: 0.66). In the osteopenia T-score subgroup, age (OR: 1.17, 95%: CI 1.13-1.21), history of parental hip fracture (OR: 3.16, 95% CI: 1.65-6.05), and glucocorticoid use (OR: 4.18, 95% CI: 1.6-11.0) were associated with ITR (Figure 4). These associations were lost in the osteoporosis T-score subgroup.

## Discussion

A total of 10% (151/1505) of individuals would have received a different treatment recommendation if only one hip was measured as part of standard DXA screening. Both the left and right hips equally did not report osteoporosis in 6% of people. Most individuals with differing treatment recommendations by the hip site had a final diagnosis of osteopenia by T-score. Age, history of parental hip fracture, and history of glucocorticoid use all had significant odds ratios associated with ITR, which should prompt measurement of the contralateral hip. These are all risk factors that are included in the FRAX calculation for treatment recommendations based on a predicted 10-year probability of fracture. In the osteoporosis T-score subgroup, however, no risk factor was associated with ITR. This means up to 12% of individuals with osteoporosis T-scores on the contralateral hip would be assessed as osteopenia not requiring treatment, and there are no clinical risk factors to prompt measurement of the contralateral hip. This highlights the importance of bilateral hip measurements to initiate medical therapy in all who would benefit from fracture risk reduction.

The ISCD states that BMD can be measured at either hip due to high concordance between left and right hip sites [3,14]. On the other hand, multiple studies have highlighted the discordance between left and right hip BMD measurements [4-7]. Of the participants, 5-8.9% had a diagnosis of osteoporosis in one hip when the contralateral hip and spine did not [4-6]. These studies relied on the least or smallest significant difference as the primary outcome. None included the FRAX calculation to determine if treatment recommendations would differ. By including the FRAX, we found a higher percentage of individuals who would not achieve the diagnosis of clinical osteoporosis requiring treatment (12% each in osteopenia and osteoporosis T-score groups, 10% overall). Calculation of FRAX is standard of care in all individuals with a T-score in the osteopenia range. In addition, all major guidelines strongly recommend treatment if there is a high 10-year probability of fracture [8-11]. The incorporation of a calculated FRAX discrepancy between hip sites is therefore prudent in determining if the measurement of both hips affects clinical care.

Age is a significant clinical factor associated with BMD discordance at the hips [4,15,16]. Like our study, age > 65 years old was positively associated with BMD discordance at the hips and this association was not influenced by sex or BMI. Race also plays a factor, with Blacks showing a higher prevalence of BMD discordance compared to Whites [5]. These clinical risk factors, in addition to those noted in our study, are determined before the DXA scan is performed via the intake form (age, parental history of hip fracture, and glucocorticoid use). The clinical flow of measuring bone densitometry at our center involves first measuring the lumbar spine. The clinical densitometrist then analyzes the spine to ensure at least two vertebrae are evaluable to calculate the T-score. If not, the distal radius is measured. Both hips are then measured. After the visit, the latter sites are evaluated to determine T-scores. To reduce the number of patients misdiagnosed due to unilateral hip measurements, the clinical densitometrist can determine if bilateral hips should be measured after the lumbar spine is evaluated; if the lumbar spine T-score is in the osteoporosis range, then only one hip site is needed. If the lumbar spine’s T-score is not in the osteoporosis range and clinical risk factors are present, both hips should be measured. A drawback to this approach would be if the individual subsequently experienced a hip fracture or underwent a hip replacement at the hip site that was chosen to be measured. This would prevent follow-up comparisons. Serial BMD measurements are utilized in clinical decision-making to determine the progression of bone loss prior to initiation of treatment (e.g., in the patient who stopped glucocorticoids), monitoring BMD while on treatment, and monitoring after treatment was stopped (e.g., drug holiday) to determine if treatment should be reinitiated. If the unilateral hip becomes unusable, serial BMD measurements would be fruitless, adversely affecting patient care.

At our institution, six healthcare centers measure BMD. At the start of this project, only one location performed bilateral hip measurements. Given that patient care was adversely affected in up to 12% of people with abnormal BMD, it was standardized across all healthcare centers to measure BMD at both hips. This change did not significantly increase appointment time or cost. Chen et al. noted a 55-second increase in scan time when both hips were measured compared to one hip [17]. Implementing a clinical flow where the bone densitometrist determines whether to measure single or bilateral hip sites would therefore not result in any overall benefit to patient care. On the other hand, routinely measuring both hips would increase radiation exposure, albeit marginally (<1 µSV for peripheral DXA and 70 µSV for whole body DXA) [18]. Given that many fractures occur in the osteopenia T-score range (fracture rate ratio: 1.8, 95% CI: 1.49-2.18) [19] and the healthcare cost of one hip fracture is more than US$50,000 per patient [15], the benefits of bilateral hip measurement likely outweigh the risks.

Major strengths of our study are the use of one DXA machine and the focus on the effect on clinical management by incorporating FRAX assessment thereby making it a clinically meaningful study. Our study, however, was not designed to determine if bilateral hip measurements result in a reduction in clinical fractures compared to unilateral hip scanning. This was a retrospective chart review at an academic health system, therefore, subject to inherent bias in the patient population screened and the limitations with electronic health record data. We also relied on FRAX measurements, which can underestimate clinical fracture risk in certain populations [20-22]. In addition, our retrospective cohort lacked diversity to determine if ITR occurs similarly across ethnicities. Lastly, this was not a cost-effective analysis and a larger study with longer follow-up would prove useful to determine if bilateral hip measurement reduces clinical fractures and is more cost-effective compared to unilateral hip measurements in preventing fractures.

## Conclusions

In conclusion, measuring BMD at both hips may lead to a change in clinical management in up to 10% of individuals (12% with osteopenia) with minimal risks associated and no additional cost. Important clinical factors that should prompt measurement of both hips are age ≥ 68 years old, history of parental hip fracture, and glucocorticoid use when the lumbar spine T-score is not in the osteoporosis range. Larger, multicenter trials are needed to determine the cost-effectiveness and if there is a meaningful fracture risk reduction with bilateral hip measurements.
